# PREDICTIVE VALUE OF THE GENERAL MOVEMENTS ASSESSMENT IN PRETERM INFANTS: A META-ANALYSIS

**DOI:** 10.1590/1984-0462/2020/38/2018286

**Published:** 2020-05-08

**Authors:** Camila da Silva Pires, Sérgio Tadeu Martins Marba, Jamil Pedro de Siqueira Caldas, Mônica de Carvalho Sanchez Stopiglia

**Affiliations:** a Universidade Estadual de Campinas, Campinas, SP, Brazil.

**Keywords:** Neurologic examination, Infant, premature, Prognosis, Cerebral palsy, Exame neurológico, Recém-nascido prematuro, Prognóstico, Paralisia cerebral

## Abstract

**Objective:**

To discuss the predictive value of the General Movements Assessment for the diagnosis of neurodevelopment disorders in preterm newborns.

**Data source:**

We conducted a systematic literature review using the following databases: Scientific Electronic Library Online (SciELO), National Library of Medicine, National Institutes of Health (PubMed), and Excerpta Medica Database (EMBASE). The articles were filtered by language, year of publication, population of interest, use of Prechtl’s Method on the Qualitative Assessment of General Movements, and presence of variables related to the predictive value. The Quality Assessment of Diagnostic Accuracy Studies 2 was used to assess the methodology of the included studies. Sensitivity, specificity, Diagnostic Odds Ratio, positive and negative likelihood ratio, and parameter of accuracy were calculated.

**Data synthesis:**

Six of 342 articles were included. The evaluation of Writhing Movements is a good indicator for recognizing cerebral palsy, as it has high values for the sensitivity and accuracy parameters. The evaluation of Fidgety Movements has the strongest predictive validity for cerebral palsy, as it has high values in all measures of diagnostic accuracy. The quality assessment shows high risk of bias for patient selection and flow and timing of the evaluation. Therefore, the scale has potential to detect individuals with neurodevelopment disorders. However, the studies presented limitations regarding the selection of subjects and the assessment of neurological outcomes.

**Conclusions:**

Despite the high predictive values of the tool to identify neurological disorders, research on the subject is required due to the heterogeneity of the current studies.

## RESUMO

**Objetivo:**

Analisar o valor preditivo da General Movements Assessment para o diagnóstico de alterações do neurodesenvolvimento em recém-nascidos pré-termo.

**Fonte de dados:**

Foi realizada uma revisão sistemática da literatura utilizando as bases de dados: Scientific Electronic Library Online (SciELO), National Library of Medicine, National Institutes of Health (PubMed) e Excerpta Medica Database (EMBASE). Os artigos foram filtrados por idioma, ano de publicação, população de interesse, utilização do Método Prechtl de avaliação e presença das variáveis relacionadas ao valor preditivo da escala. O *Quality Assessment of Diagnostic Accuracy Studies 2* foi utilizado para avaliar a metodologia dos artigos*.* Foi realizado o cálculo de sensibilidade, especificidade, *Diagnostic Odds Ratio*, razão de verossimilhanças positiva e negativa e parâmetro de acurácia.

**Síntese dos dados:**

Foram incluídos seis artigos dentre os 342 encontrados. A escala, quando realizada no período Writhing Movements, possui bom poder discriminativo para o desfecho paralisia cerebral, com valores elevados de sensibilidade e acurácia. Quando realizada no período Fidgety Movements, possui maior valor preditivo para paralisia cerebral, com valores elevados em todas as medidas de acurácia diagnóstica. O risco de viés foi considerado elevado na seleção de pacientes e no fluxo e momento da avaliação. Desse modo, a escala tem potencial para detectar indivíduos que evoluíram com alterações do neurodesenvolvimento, porém, os artigos apresentaram limitações quanto à seleção dos sujeitos e à forma de avaliação do desfecho neurológico.

**Conclusões:**

Apesar dos altos valores preditivos descritos para identificação de alterações neurológicas, novas pesquisas são necessárias, devido à heterogeneidade dos estudos e ao método de avaliação a longo prazo do neurodesenvolvimento.

## INTRODUCTION

The survival of increasingly premature newborns has become a matter of concern, as preterm infants are known to have an increased risk for neurodevelopmental disorders.^1^ The incidence of morbidity, including cerebral palsy (CP), remains high and worrisome.^2^ ^,^ ^3^ ^,^ ^4^ ^,^ ^5^

Early diagnosis of neurodevelopmental changes is essential for planning interventions that promote adequate growth and development of preterm infants, attenuate complications resulting from brain injury, and improve the child’s future functionality. However, early diagnosis still represents a major clinical challenge.^4^ ^,^ ^5^ ^,^ ^6^ ^,^ ^7^ ^,^ ^8^

The application of neuromotor or neurobehavioral assessment scales can be performed as a predictive instrument. Some factors may influence the performance of preterm infants when they undergo certain tests, as they are clinically fragile and may be unable to maintain sufficient energy reserves to produce the best results throughout the evaluation.^6^ ^,^ ^9^ ^,^ ^10^

Taking into consideration these factors, the General Movements Assessment (GMA), which has not yet been translated into Portuguese, is indicated for fragile newborns, as it is a fast and non-invasive method, based on the qualitative observation and evaluation of a filmed video of the patient’s spontaneous movements.^5^ ^,^ ^6^ ^,^ ^11^ ^,^ ^12^

The observation of spontaneous movements is performed in order to determine the integrity of the central nervous system (CNS), because the quality of the movements is modulated by corticospinal or reticulo-spinal pathways and may be affected by changes in these structures. The predictive value of GMA in relation to late neurological performance is higher when compared to neurological examination based on tone assessment, primitive reflexes and the presence of postural disorders and a transfontanellar ultrasound.^2^ ^,^ ^3^ ^,^ ^4^ ^,^ ^5^ ^,^ ^12^ ^,^ ^13^ ^,^ ^14^ ^,^ ^15^ ^,^ ^16^ ^,^ ^17^ ^,^ ^18^ ^,^ ^19^ ^,^ ^20^

GMA is a standardized functional assessment of the CNS and allows for the observation of spontaneous movement of the newborn.^12^ Movements are complex, occur frequently, and are long enough to be properly observed. They are classified by Prechtl according to age, and are called: fetal and preterm movements up to 40 weeks of gestational age; Writhing Movements (WM), present from 40 weeks of gestational age to the ninth week post term; Fidgety Movements (FM), present from week 9 to week 20 post-term.^3^ ^,^ ^12^ ^,^ ^14^ ^,^ ^17^ ^,^ ^18^

As for fetal and preterm movements, the generalized movements presented by the fetus and preterm infants show practically no difference, indicating that both the increase in the force of gravity after birth and maturation have no influence on their onset.^12^ ^,^ ^17^ ^,^ ^18^ ^,^ ^21^

WM are characterized by a small to moderate amplitude and low to moderate speed. The movements occur in elliptical shapes, which gives the impression of writhing. Such movements involve the whole body in a variable sequence of upper and lower limbs, the neck, and the torso. They increase and decrease in intensity, strength and speed, have a gradual beginning and end, and rotate along the axis of the limbs. Slight changes in direction of movement make them fluid and create the impression of complexity and variability.^3^ ^,^ ^12^ ^,^ ^14^ ^,^ ^17^ ^,^ ^18^ ^,^ ^21^

From the fetal period to the ninth week postpartum, abnormal patterns are classified into the following categories:^12^ ^,^ ^14^ ^,^ ^19^ poor repertoire (PR) - motor patterns with a monotonous sequence and a complexity that is different from normal; cramped synchronized (CS) - rigid movements that do not flow and do not have the elegance and complexity that are characteristic of normal patterns (limb and trunk muscles contract and relax simultaneously); chaotic (CA) - large amplitude movements, devoid of the fluidity and elegance of regular motor patterns.

FM are characterized by low-limb, torso and head movements, moderate speed, variable acceleration, and small hand and foot rotation movements that create an elegant look. They are present continuously while the child is awake, except during visual fixation. With the onset of voluntary movements, irregular movements become less expressive, but are still present while the infant sleeps, up to six months of age.^3^ ^,^ ^12^ ^,^ ^14^ ^,^ ^17^ ^,^ ^18^ ^,^ ^21^ The abnormal patterns in this period are classified into the following categories:^12^ ^,^ ^14^ ^,^ ^19^ absent - no irregular movements; abnormal - moderate or severe increase in amplitude and velocity, and irregular movements are no longer continuous.

Since preterm infants have a high risk of brain injury, the use of predictive scales may enable early detection of neurodevelopmental disorders, and because the GMA scale can be adequately applied to preterm infants, the aim of this study was to evaluate the predictive value of GMA for the detection of neurodevelopmental disorders in preterm infants.

## METHOD

A systematic literature review was performed from January to February 2018, using the following databases: Scientific Electronic Library Online (SciELO), National Library of Medicine, National Institutes of Health (PubMed) and Medical Excerpt dataBASE (EMBASE)*.* The population of interest was preterm newborns and, as an intervention, the Prechtl method of assessing generalized movements - GMA - was used. Descriptors were selected considering the fact that the GMA scale is not translated into Portuguese. Thus, the following English language terms were used: “general movements” and “preterm infant” in association with the terms, “prediction”, “neurological outcome”, “predict validity” and “sensitivity”.

Inclusion criteria were: articles in the English or Portuguese language, published in the last ten years, that had the term “General Movements” in the title, abstract or keywords. Eligibility criteria were: longitudinal descriptive or observational clinical studies in which the population evaluated was only preterm infants evaluated within the corrected 40-week period up to 20 weeks post-term; studies that related the application of the test to neurological evolution at 12 months or more of the corrected age, and studies using the Prechtl method of movement assessment. Exclusion criteria were: review articles (integrative or systematic or meta-analysis), abstracts published at events or poster presentations, editorials, and articles published in full that did not describe the predictive values of sensitivity, specificity, positive predictive value (PPV) and negative predictive value (NPV).

First, the studies were selected by reading the titles and keywords, based on the inclusion criteria established. Subsequently, a detailed reading of the abstracts was performed, and then articles whose abstracts did not meet the eligibility criteria were excluded. After the refinement of the texts, the articles were read in full and the exclusion criteria were applied. Finally, the included articles were cataloged according to their characteristics, risk of bias and results. When the information pertinent to the sample was incomplete, the authors were contacted by email. From the data, the statistical analysis was performed. Results and discussion were presented descriptively.

This systematic review was registered on the PROSPERO platform (International Prospective Register of Systematic Reviews). In order to ensure the quality of the work, the literature search and methodological analysis of each article were performed by two authors independently, according to the Quality Assessment of Diagnostic Accuracy Studies 2 (QUADAS-2). In case of disagreement, the authors reevaluated the articles until they reached a consensus. A quality analysis of the clinical studies was performed with the aid of QUADAS-2, and articles were evaluated for methodological criteria regarding patient selection, for the test under evaluation (index test), for the reference test (gold standard), and for the flow and timing of the assessment. They were classified as having low, high and uncertain risk of bias.

An exploratory analysis was performed by calculating sensitivity, specificity, the Diagnostic Odds Ratio (DOR), positive likelihood ratio (LR +) and negative likelihood ratio (LR-). To assess the homogeneity of sensitivities and specificity, the chi-square test was applied. Only the univariate approach was applied because there was a small number of articles. The accuracy parameter (θ) was estimated using the Proportional Hazards Model (PHM).^22^

## RESULTS

### Description of the articles included

A total of 342 publications were found, distributed among the SciELO (n = 99), PubMed (n = 138) and EMBASE (n = 105) databases. After the duplicate deletion of 233 articles, 109 were selected. From these, the following were removed: three for being written in different languages (Chinese, Spanish and French); 29 for having been published prior to the period defined in the inclusion criteria; and seven for not containing “General Movements” in their title, summary or keywords.

At this stage, 70 abstracts were read carefully, and the eligibility criteria were applied. Twenty-eight articles were excluded because their objective was to describe the scale, or to evaluate the electronic program, or they related the scale with some type of intervention. Thirteen were excluded for not evaluating preterm infants.

Of the 29 remaining articles, all were read in full, and the exclusion criteria were applied. Thus, 11 texts were excluded because they dealt with systematic reviews, abstracts or letters to the editor, 10 were excluded because they did not differentiate between all of the predictive values of the test, and 2 were excluded because they used the Hadders-Algra method of movement evaluation. Finally, six articles met all of the criteria and were included in this paper.^23^ ^,^ ^24^ ^,^ ^25^ ^,^ ^26^ ^,^ ^27^ ^,^ ^28^ Their characterization by author, year, study design, time of GMA assessment, age of final assessment, rating scale, and outcome of neurological outcome is shown in Table 1.

**Table 1.** Characterization of the articles included according to author, year, study design, timing of the General Movements Assessment, age of the final assessment, rating scale and outcome of the neurological evolution.

Author, year	Study Design	GMA Evaluation Time	Final evaluation (months)	Rating Scale	Outcome
Olsen et al.^23^	Longitudinal descriptive clinical trial	WM	12	AIMS, NSMDA, TINE	Neurological dysfunction
Dimitrijevic et al.^24^	Longitudinal descriptive clinical trial	WM FM	24	TINE	CP
Spittle et al.^25^	Longitudinal descriptive clinical trial	WM FM	24, 48	BAYLEY MABC-2 DAS-II	CP
Sustersic et al.^26^	Longitudinal descriptive clinical trial	WM FM	60-72	M-ABC	Neurological dysfunction
Burger et al.^27^	Longitudinal descriptive clinical trial	FM	12	PDMS-II AIMS	PC
Spittle et al.^28^	Longitudinal descriptive clinical trial	WM FM	12	AIMS, NSMDA	Neurological dysfunction CP

WM: Writhing Movements; FM: Fidgety Movements; GMA: General Movements Assessment; CP: cerebral palsy; AIMS: Alberta Infant Motor Scale; NSMDA: Neurological, Sensory, Motor, Developmental Assessment; TINE: Touwen Infant Neurological Examination; MABC-2: Movement Assessment Battery for Children-Second Edition; DAS-II: Differential Ability Scale-Second Edition; PDMS-II: Peabody Developmental Motor Scale II; BSID-II: Bayley Scales of Infant Development II.

The articles showed differences in when the GMA was performed, the scales used for the diagnosis of neurodevelopmental sequelae and the long-term outcome of neurological evolution. One article applied GMA during the corrected 40-week WM period,^23^ one article applied GMA during the 12-week postpartum FM period^27^, and four performed the evaluation in both periods, four and 12 weeks of age post-term.^24^ ^,^ ^25^ ^,^ ^26^ ^,^ ^28^ Three articles assessed late neurological performance at 12 months of corrected age,^23^ ^,^ ^27^ ^,^ ^28^ one at 24-months of corrected age,^24^ one at 24 and 48 months^25^ and one at 60 and 72 months postnatal age.^26^ Different scales were applied in the final assessments of the neurological outcome (Table 1).

Although four studies included in this review adopted the presence or absence of CP in the long-term outcome of neurological outcome^24^ ^,^ ^25^ ^,^ ^27^ ^,^ ^28^, three articles discuss the predictive value of GMA for minor neurological dysfunctions.^23^ ^,^ ^26^ ^,^ ^28^ They are characterized by mild motor, sensory, and or cognitive changes and may be called minimal brain dysfunction, apraxia, dyspraxia, integrative sensory dysfunction, or coordination disorder.

Therefore, due to methodological differences, the variables were analyzed according to the moment of GMA assessment (WM or FM) and the long-term outcome (CP or minor neurological dysfunction).

### Quality of the articles included

Of the total articles evaluated, 83.3% had a high risk of bias in patient selection. This result was due to the lack of clarity about the mode of selection of the subjects; the use of inappropriate exclusion criteria; and the selection of participants, who were screened in rehabilitation centers and already had some risk factor for neuromotor developmental delay in their medical history. Regarding the evaluation of how the test was performed, in the case of GMA, and of the reference standard, 100% of the articles presented a low risk of bias. The flow analysis and the evaluation timing showed a 16.7% risk of high bias. One article described the failure to perform outcome assessment in all patients and the lack of formal assessment for the diagnosis of CP as limitations of the study.^23^

### Result from the meta-analysis: writhing movements versus cerebral palsy

**Figure 1.**
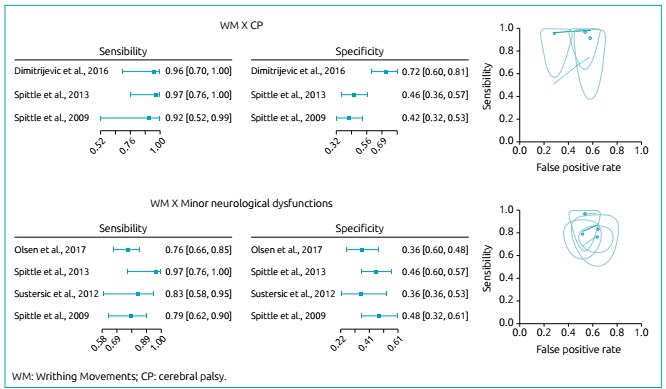
Forest plot, SROC curve, observed data, and reliable ellipse for observations in the Writhing Movements period.

We considered three articles that described the relationship between the assessment in the WM period and the outcome of CP. ^24^ ^,^ ^25^ ^,^ ^28^ The total number of subjects was 264. The mean gestational age and weight were 27.3 weeks and 1,011 g, respectively. However, one article did not provide the mean gestational age and birth weight values (Table 2).^24^ The GMA sensitivity forest-plot graphs of the three studies for CP diagnosis in the WM period are depicted in Figure 1. The sensitivity, specificity, LR +, LR-, and DOR values of each article are described in Table 2. The Chi-square test showed heterogeneous sensitivity (p=0.868) and homogeneous specificity (p<0.001). The estimated accuracy parameter considering heterogeneity was 0.030 (95%CI 0.00‒0.53) and considering the area under the curve, 0.971 (95%CI 0.656‒1.000).

**Table 2.** Analysis of the General Movements Assessment to predict cerebral palsy and minor neurological dysfunctions in the Writhing Movements period.

Outcome	Article	n	Age Average	Weight Average	SENS 95%CI	ESP 95%CI	DOR 95%CI	LR + 95%CI	LR- 95%CI
CP	Dimitrijević et al.^24^	79	NF	NF	0.958 (0.699-0.996)	0.717 (0.602-0.810)	58.385 (3.28-1039.6)	3.391 (2.287-5.029)	0.058 (0.004-0.879)
	Spittle et al.^25^	99	27.3 ± 1.5	1008 ± 265	0.969 (0.759-0.997)	0.465 (0.363-0.570)	26.912 (1.56-464.43)	1.810 (1.457-2.248)	0.067 (0.004-1.039)
	Spittle et al.^28^	86	27.3 ± 1.5	1014 ± 265	0.917 (0.517-0.991)	0.421 (0.320-0.529)	7.989 (0.43-149.34)	1.582 (1.168-2.144)	0.198 (0.014-2.848)
Minor Neurological dysfunctions	Olsen et al.^23^	137	27.8±1.5	1031 ± 262	0.764 (0.655-0.846)	0.362 (0.256-0.483)	1.828- (0.875-3.821)	1.196 (0.957-1.494)	0.654 (0.388-1.102)
	Spittle et al.^25^	99	27.3 ± 1.5	1008 ± 265	0.969 (0.759-0.997)	0.465 (0.363-0.570)	26.912 (1.56-464.43)	1.810 (1.457-2.248)	0.067 (0.004-1.039)
	Sustersic et al.^26^	45	31.6 ± 3.3	1788 ± 718	0.833 (0.584-0.947)	0.359 (0.217-0.532)	2.805 (0.60-13.06)	1.301 (0.922-1.836)	0.464 (0.137-1.575)
	Spittle et al.^28^	86	27.3 ± 1.5	1014 ± 265	0.790 (0.619-0.897)	0.482 (0.358-0.609)	3.514 (1.281-9.636)	1.527 (1.121-2.081)	0.435 (0.209-0.906)

CP: cerebral palsy; n: sample; NF: not informed; SENS: sensitivity; ESP: specificity; 95%CI: 95% confidence interval; DOR: Diagnostic Odds Ratio; LR +: positive likelihood ratio; LR-: negative likelihood ratio.

### Result from the meta-analysis: writhing movements versus minor neurological dysfunctions

We considered four articles that described the relationship between WM assessment and the outcome of minor neurological dysfunctions. The total number of subjects was 367; the mean gestational age and weight were 28.5 weeks and 1,210.3 g, respectively (Table 2).^23^ ^,^ ^25^ ^,^ ^26^ ^,^ ^28^ The GMA sensitivity and specificity forest-plot graphs of the four studies for diagnosis of minor neurological dysfunctions in the WM period are depicted in Figure 1. The sensitivity, specificity, LR +, LR- and DOR values of each article are described in Table 2. The Chi-square test showed heterogeneous sensitivity (p=0.308) and homogeneous specificity (p=0.400). The estimated accuracy parameter considering heterogeneity was 0.313 (95%CI 0.054‒0.571) and considering the area under the curve, 0.762 (95% CI 0.637‒0.948).

### Result from the meta-analysis: writhing movements versus cerebral palsy

**Figure 2.**
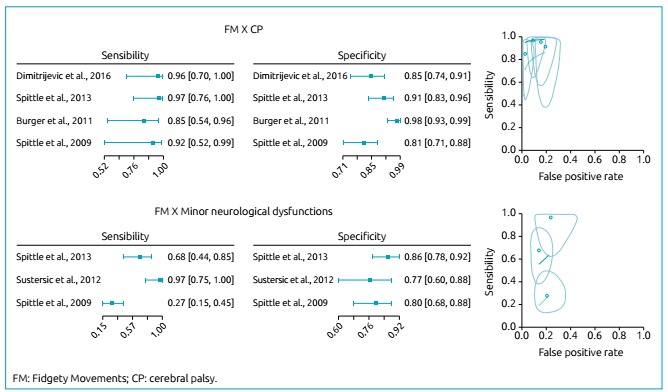
Forest plot, SROC curve, observed data, and reliable ellipse for observations in the Fidgety Movements period.

We considered four articles ^24^ ^,^ ^25^ ^,^ ^27^ ^,^ ^28^ that described the relationship between assessment during the FM period and the outcome of CP. The total number of subjects was 379; the mean gestational age and weight were 28.2 weeks and 1,020.3 g, respectively. However, one article^24^ did not provide the mean gestational age and birth weight values (Table 3). The GMA sensitivity and specificity forest-plot graphs of the four studies for the diagnosis of CP in the FM period are depicted in Figure 2. The sensitivity, specificity, LR +, LR- and DOR values of each article are described in Table 3. The Chi-square test showed heterogeneous sensitivity (p = 0.670) and homogeneous specificity (p=0.001). The estimated accuracy parameter considering heterogeneity was 0.013 (95% CI 0.00‒0.09) and considering the area under the curve, 0.987 (95% CI 0.920‒1.000).

**Table 3.** Analysis described of the General Movements Assessment to predict cerebral palsy and minor neurological dysfunctions in the Fidgety Movements period.

Outcome	Article	n	Age Average	Weight Average	SENS 95%CI	ESP 95%CI	DOR 95%CI	LR + 95%CI	LR- 95%CI
CP	Dimitrijević et al.^24^	79	NF	NF	0.958 (0.699-0.996)	0.848 (0.745-0.914)	128.143 (7.004-2344.4)	6.298 (3.564-11.128)	0.049 (0.003-0.743)
	Spittle et al.^25^	99	27.3 ± 1.5	1008 ± 265	0.969 (0.759-0.997)	0.912 (0.832-0.956)	320.333 (17.36-5905.0)	10.979 (5.512-21.868)	0.034 (0.002-0.525)
	Burger et al.^27^	115	30 ± 2.1	1039.30 ± 160.6	0.850 (0.541-0.965)	0.977 (0.928-0.993)	236.867 (27.83-2016.4)	36.380 (10.4-127.3)	0.154 (0.035-0.672)
	Spittle et al.^28^	86	27.3 ± 1.5	1014 ± 265	0.917 (0.517-0.991)	0.811 (0.713-0.881)	47.194 (2.477-899.3)	4.849 (2.915-8.069)	0.103 (0.007-1.463)
Minor neurological dysfunctions	Spittle et al.^25^	99	27.3 ± 1.5	1008 ± 265	0.676 (0.440-0.847)	0.865 (0.776-0.922)	13.364 (4.061-43.977)	5.000 (2.663-9.388)	0.374 (0.187-0.748)
	Sustersic et al.^26^	45	31.6 ± 3.3	1788 ± 718	0.967 (0.747-0.997)	0.766 (0.596-0.879)	94.733 (5.031-1783.9)	4.124 (2.190-7.769)	0.044 (0.003-0.669)
	Spittle et al.^28^	86	27.3 ± 1.5	1014 ± 265	0.274 (0.149-0.449)	0.798 (0.677-0.882)	1.495 (0.539-4.147)	1.359 (0.629-2.939)	0.909 (0.706-1.171)

CP: cerebral palsy; n: sample; NF: not informed; SENS: sensitivity; ESP: specificity; 95%CI: 95% confidence interval; DOR: Diagnostic Odds Ratio; LR+: positive likelihood ratio; LR-: negative likelihood ratio.

### Result from the meta-analysis: writhing movements versus minor neurological dysfunctions

We considered four articles that described the relationship between FM assessment and the outcome of minor neurological dysfunctions. The total number of subjects was 230; the mean gestational age and weight were 28.7 weeks and 1,270 g, respectively (Table 3).^25^ ^,^ ^26^ ^,^ ^28^ The GMA sensitivity and specificity forest-plot graphs of the three studies for the diagnosis of minor neurological dysfunctions in the FM period are depicted in Figure 2. The sensitivity, specificity, LR +, LR- and DOR values of each article are described in Table 3. The Chi-square test showed homogeneous sensitivity (p<;0,001) and heterogeneous specificity (p=0,370). The estimated accuracy parameter considering heterogeneity was 0.30 (95% CI 0.00‒0.83) and considering the area under the curve, 0.770 (95% CI 0.545‒1.000).

## DISCUSSION

Novak et al. emphasize the importance of diagnosing neuromotor development disorders early in order to optimize cognitive and motor plasticity, as well as to prevent secondary complications in children with CP. Therefore, a combination of predictive diagnostic tools, such as clinical history, magnetic resonance imaging and GMA is recommended.^29^ ^,^ ^30^ Other systematic reviews point to GMA as the scale that is most associated with long-term neurological outcomes compared with other scales.^6^ ^,^ ^9^ The findings of the present meta-analysis corroborate such work and suggest that the scale may provide important information about the evolution of preterm infants, especially regarding the diagnosis of CP.

Analysis of the predictive outcome of GMA performed up to the ninth week post-term age (WM) for the CP outcome showed high sensitivity values; therefore, GMA has the potential of being effective in detecting individuals who will evolve with CP. However, the specificity values were low, ie, the change in assessment did not necessarily reflect future changes in neurodevelopment. The analysis of the ratio between the probability of a positive result in individuals with an alteration and the probability of a positive result in individuals without an alteration (DOR) also suggested that the scale is a good tool to help diagnose CP, since two articles presented elevated values from this index (26,912 and 58,385). The LR+ indicates how much an altered test result influences an individual’s chances of actually having the alteration. Its value varies from one to infinity, and the higher the number, the better the ability of the test to identify the individual with the alteration. The LR- indicates how much an unchanged test result influences the individual’s chance of not actually having the alteration. Its value ranges from 0 to 1, and the smaller the number, the greater the ability of the test to identify an individual without an alteration. An analysis of such diagnostic accuracy measures was also performed. The LR + values suggested that the test may be considered useful in identifying individuals with alterations, while those from the LR- suggested that the test may also be considered useful in identifying individuals who have evolved without CP. An accuracy parameter and an area under the curve analysis also suggested that the test has good discriminative power, as the area under the curve values greater than 0.8 indicate that the test is very accurate.

Still in the WM period, the analysis of the GMA predictive result for minor neurological dysfunctions showed lower sensitivity and specificity values. The LR +, the LR-, the accuracy parameter, and the area under the curve confirmed the ability of the test to identify the alteration, however it was not very accurate when finding individuals that did and did not have developmental alterations. Only the article by Spittle et al. presented higher values of sensitivity, LR- and DOR, but with upper and lower limits far from the 95%CI.^25^

Analysis of the predictive outcome of the GMA performed from the ninth to the twentieth week of post-term age (FM) for CP outcome showed high sensitivity and specificity values. This result suggests that this time interval is ideal for performing GMA to predict CP, that is, the odds of an individual with CP having their test altered and another without CP having their test normal are high. The LR+ values found reflect moderate to optimal accuracy. In two articles, the ability of the test to identify the individual with an alteration was optimal. The values of the LR- suggest from moderate to excellent accuracy, that is, most individuals who evolved without CP presented a normal result in the evaluation. DOR values suggested that GMA, when performed during this period, is an important tool for the diagnosis of CP. The accuracy parameter and the area under the curve suggested that the test, when performed in the FM period, is more accurate, and the values confirmed higher sensitivity and fewer cases of false positives.

An analysis of the predictive outcome of GMA in the FM period for minor neurological dysfunctions after 12 months of corrected age showed relatively low values for all of the parameters analyzed. Thus, the result indicates that the analysis of the GMA result during this period does not have a high predictive value. Only the article by Sustersic et al. presented higher values of sensitivity, LR+ LR-, and DOR, however its upper and lower limits were far from the 95%CI.

One stduy^30^ suggests that the relationship of GMA with other neurological disorders, mainly cognitive, seems to be associated not only with the presence of normal movements in this period, but rather with the time when the normalization of the evaluation occurred, ie, it is related to the child’s trajectory over time.

The predictive values of the CP scale, especially in the FM period, were quite high, while the relationship of GMA with other late neurodevelopmental dysfunctions still deserves further investigation. However, the generalization of the data presented for the preterm population is limited by two factors. The first is related to the high risk of selection bias of the participants of the evaluated articles, because the samples were composed of preterm infants who had a higher risk for neurodevelopmental disorders and were at rehabilitation centers. This is true in all but one article, in which the selection of the subjects happened consecutively in the Neonatal Intensive Care Unit (NICU).^27^ The second factor is related to the heterogeneity of the articles, since the upper and lower confidence interval limits (95% CI) were distant. This fact may be related to the use of different scales to evaluate late neuromotor development.

Other systematic review articles^6^ ^,^ ^31^ ^,^ ^32^ ^,^ ^33^ ^,^ ^34^ and meta-analysis^13^ on the predictive value of the GMA have been previously published, but they have not exclusively selected the preterm infant population or they have not discussed the heterogeneity and risk of bias as presented in this paper. Nonetheless, the present work has some limitations that deserve attention: an analysis limited to studies published only in English and Portuguese; a small number of articles included; gestational age and weight averages were not present in one of the included studies; and outcome measures varied among articles, resulting in heterogeneity of sensitivity and specificity values, precluding accuracy in the meta-analysis.

It can be concluded that, despite the high predictive values described by GMA for the identification of neurological alterations (especially in the FM period), the publication of new studies is necessary due to the heterogeneity of the studies and the long-term nature of the neurodevelopmental evaluation method. The translation and validation of the scale into Portuguese would encourage its use in clinical practice and, consequently, the publication of new studies in our country, complementing findings that have already been disclosed.
